# Comparison of early *versus* late addition of granulocyte and monocyte adsorption for incomplete remission induction in ulcerative colitis

**DOI:** 10.1002/jgh3.70012

**Published:** 2024-07-24

**Authors:** Keiichi Tominaga, Mimari Kanazawa, Shoko Watanabe, Takanao Tanaka, Shunsuke Kojimahara, Satoshi Masuyama, Keiichiro Abe, Akira Kanamori, Akira Yamamiya, Takeshi Sugaya, Kenichi Goda, Yuji Fujita, Shigemi Yoshihara, Yasuo Haruyama, Atsushi Irisawa

**Affiliations:** ^1^ Department of Gastroenterology Dokkyo Medical University School of Medicine Tochigi Japan; ^2^ Department of Pediatrics Dokkyo Medical University School of Medicine Tochigi Japan; ^3^ Integrated Research Faculty for Advanced Medical Sciences Dokkyo Medical University Tochigi Japan

**Keywords:** granulocyte and monocyte adsorption, incomplete, remission induction therapy, ulcerative colitis

## Abstract

**Background and aim:**

Ulcerative colitis (UC) is characterized by repeated relapse and remission. Because no fundamental therapeutic strategy has been established, the treatment goal is generally to maintain the remission phase for a long period after rapid remission induction. Granulocyte and monocyte adsorption (GMA) for UC is reportedly quite safe because it does not affect immunosuppression. Moreover, it is useful in combination with other remission induction therapy. The aim of this study was to evaluate the difference in efficacy by the timing of the addition of GMA with corticosteroids, calcineurin inhibitors, and anti‐cytokine therapy for active UC.

**Methods:**

The study included 59 patients. Patients who started GMA of 5–11 days were in the early GMA combination group. Patients who started GMA 12 days or more were in the late GMA combination group. The primary endpoint was difference in the effect of additional GMA according to the timing of the intervention. The secondary endpoint was difference in the time to remission induction between the two groups.

**Results:**

Of the 32 early GMA group patients, 24 achieved remission induction. Of the 27 late group patients, 18 achieved remission induction. No significant difference in induction rates was found (*P* = 0.481). The early group had shorter mean time to remission induction (*P* < 0.001).

**Conclusions:**

In conclusion, results suggest that early addition of GMA might lead to earlier remission in patients who have had an inadequate response to remission induction therapy with corticosteroids, calcineurin inhibitors, and anti‐cytokine therapy.

## Graphical abstract

Early addition of granulocyte and monocyte adsorption might lead to earlier remission in patients who have had an inadequate response to remission induction therapy with corticosteroids, calcineurin inhibitors, and anti‐cytokine therapy.

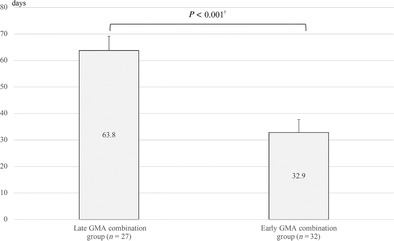

## Introduction

Ulcerative colitis (UC) is a chronic inflammatory bowel disease (IBD) with recurrent symptoms. Because its precise etiology remains unknown, treatment emphasizes the management of inflammation using pharmacological agents.^1^ When disease activity persists after administration of mesalamine, corticosteroids are the first‐line agents for remission induction therapy. If remission induction is difficult to achieve with corticosteroids, then calcineurin inhibitors or anti‐cytokine therapy such as anti‐tumor necrosis factor (TNF)‐α antibodies might be considered.^2^, ^3^ Nevertheless, no remission induction therapy has been found to provide 100% efficacy. For all treatments, many patients show inadequate or ineffective response.^4^, ^5^, ^6^, ^7^, ^8^, ^9^, ^10^, ^11^, ^12^, ^13^, ^14^, ^15^

In the early 2000s, granulocyte and monocyte adsorption (GMA) was introduced into the Japan national health reimbursement scheme as remission induction therapy for patients with UC. In actuality, GMA treatment removes granulocytes and monocytes from the patient's blood. Unlike corticosteroids, calcineurin inhibitors, and anti‐cytokine therapies, GMA is reported to be quite safe because it is a nonimmunosuppressive therapy.^16^, ^17^ The efficacy of GMA after 7 weeks in a clinical trial was reported as 58.5% for GMA monotherapy.^18^ Furthermore, several subsequent reports have described efficacy rates exceeding 70% with the introduction of intensive therapy and increased blood volume to be treated.^19^, ^20^, ^21^ Real‐world data of GMA monotherapy in 656 patients indicate that only 7.7% of patients had mild to moderate adverse events (AEs) (1.6% headache, 1.3% fever/chills) and no severe AE.^22^ Because its therapeutic mechanism differs from those of other agents, GMA is useful in combination with corticosteroids, calcineurin inhibitors, and anti‐cytokine therapy. Several reports have described the efficacy and safety of combination therapy with GMA and anti‐cytokine therapy, but no report has described a study assessing the efficacy of adding GMA after starting remission induction therapy, or a study of the appropriate timing for adding GMA.

The main purpose of this study was to evaluate the difference in efficacy by timing of the addition of GMA to remission induction therapy with corticosteroids, calcineurin inhibitors, and anti‐cytokine therapy for active UC.

## Methods

### Study design

The ethics committee of Dokkyo Medical University Hospital approved this retrospective study (approval no. R‐51‐1J). This study was conducted in accordance with the ethical principles outlined in the Declaration of Helsinki and registered in the University Hospital Medical Network Clinical Trials Registry (UMIN000049168). The ethics committee of the Dokkyo Medical University Hospital deemed, because of the study's retrospective nature, that written informed consent was replaceable by the obligation of informing participants and giving participants the right and opportunity to opt out. We provided participants a means to opt out, instead of using informed consent, which safeguarded opportunities for research subjects to notify and publish research information related to our website. The option to opt out of the study was communicated to participants via our website with the following message. “Dokkyo Medical University Hospital is now conducting research using medical data from patients treated for ulcerative colitis. There will be no additional burden on patients for conduct of this study. Additionally, we will conduct the research in compliance with laws and regulations related to the protection of patient privacy. If you do not want your medical data to be used for this study, please contact your doctor.”

The primary endpoint of this study was the difference in the effect of additional GMA according to the timing of the intervention. The secondary endpoint was the difference in the time to remission induction between the early and late GMA combination groups.

### Patient characteristics and treatment strategies of UC

**Figure 1:**
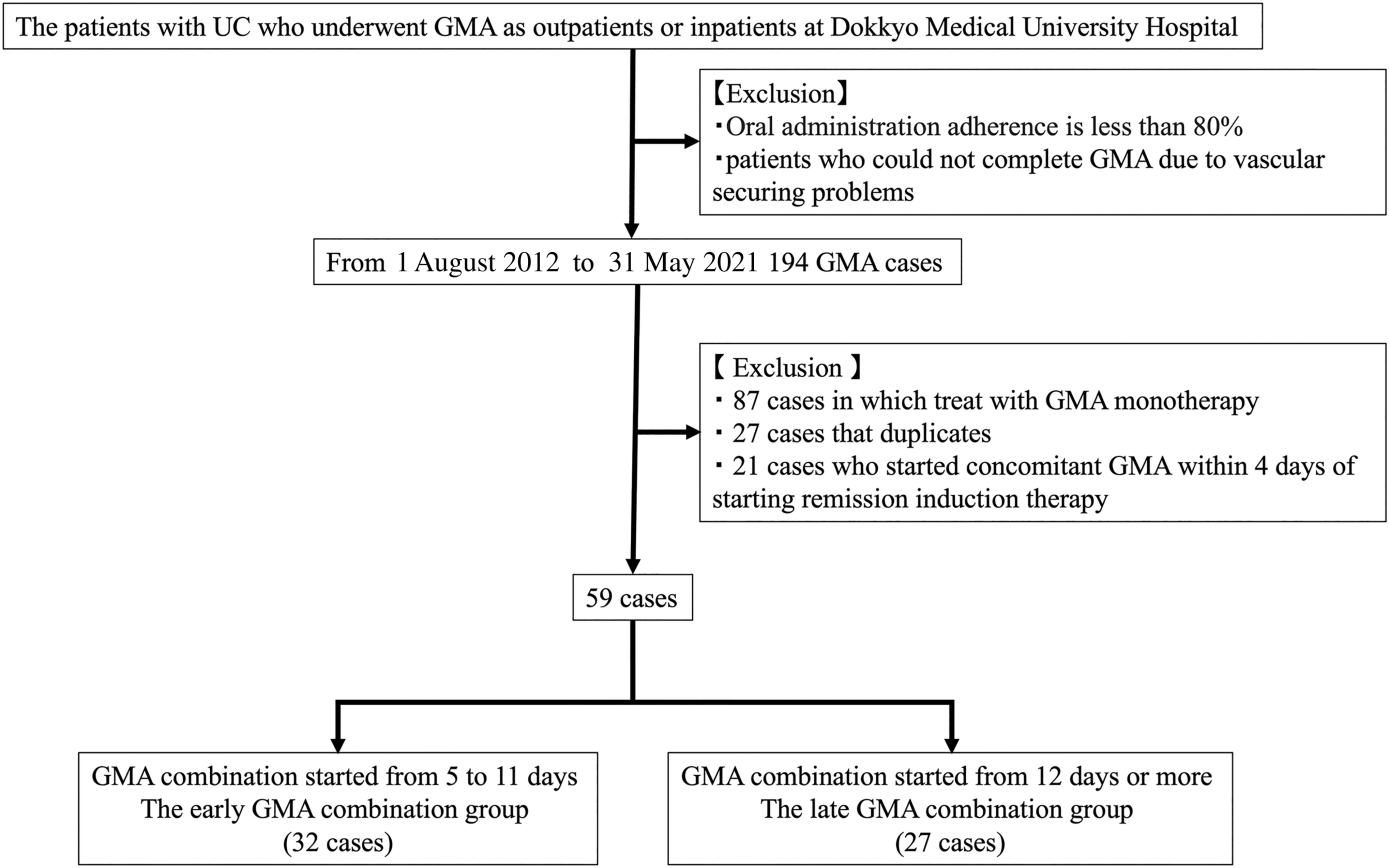
Patient inclusion and exclusion criteria. GMA, granulocyte and monocyte adsorption; UC, ulcerative colitis.

Table 1 presents the baseline characteristics of this study. At our institution, induction of remission therapy for active UC is performed in accordance with the Japanese evidence‐based clinical practice guidelines for IBD.^3^, ^23^ To select eligible patients, we reviewed medical records retrospectively. Patients with UC who underwent GMA as outpatients or inpatients at Dokkyo Medical University Hospital were included in this study. Patients whose adherence to oral medications was less than 80% of the prescribed dose based on medical record entries and patients who were unable to complete GMA because of difficulties securing vascular access were excluded from the study. During 1 August 2012, through 31 May 2021, 194 GMA cases were treated. Among them, 27 cases were duplicates. They were excluded from this study. Furthermore, 87 cases for which GMA monotherapy was used as remission induction treatment were excluded. In addition, 21 patients who started concomitant GMA within 4 days of starting remission induction therapy were excluded, considering that a minimum period of 4 days is necessary to start remission induction therapy and to ascertain whether the treatment is effective. Finally, 59 patients were included in this study (Fig. 1). For the 59 patients included in this study, the median time from the start of induction therapy to the start of GMA was 11 days. The 59 patients were divided into two groups. Those who started GMA from 5 to 11 days were in the early GMA combination group. Those who started GMA 12 days or more were in the late GMA combination group. This study was conducted from the start of remission induction therapy to 52 weeks.

**Table 1:** Background characteristics of the patients.

Characteristics (*n* = 59)	
Age (years)	38 (18–75)
Sex (male)	54.2% (*n* = 32)
Extent of disease	
Proctitis type	1.7% (*n* = 1)
Left‐sided type	13.6% (*n* = 8)
Pancolitis type	84.7% (*n* = 50)
Clinical course classification	
First attack type	6.8% (*n* = 4)
Relapse‐remitting type	89.8% (*n* = 53)
Chronic continuous type	3.4% (*n* = 2)
Disease duration (months)	34 (1–462)
Lichtiger index at the beginning of remission induction	13 (5–19)
Remission‐inducing drugs	
5‐aminosalicylic acid	23.7% (*n* = 14)
Corticosteroid	59.3% (*n* = 35)
Calcineurin inhibitor	11.9% (*n* = 7)
Anti‐cytokine therapy	5.1% (*n* = 3)
Concomitant local therapy	27.1% (*n* = 16)
PSL dependence	35.6% (*n* = 21)
PSL resistance	22.0% (*n* = 13)
5‐aminosalicylic acid intolerance	1.7% (*n* = 1)
Immunomodulator intolerance	16.9% (*n* = 10)
Clinical severity at the beginning of remission induction therapy	
Mild	0% (*n* = 0)
Moderate	74.6% (*n* = 44)
Severe	23.7% (*n* = 14)
Fulminant	1.7% (*n* = 1)
Lichtiger index at the time of GMA induction	8 (4–15)
Success rate of remission induction	71.2% (*n* = 42)

GMA, granulocyte and monocyte adsorption.

### Granulocyte and monocyte adsorption procedure

For these patients, GMA was performed using Adacolumn (Jimro Co., Ltd., Takasaki, Japan) filled with cellulose acetate beads as adsorptive carriers that selectively remove granulocytes and monocytes (Fc‐gamma‐R and complement receptors bearing leukocytes); lymphocytes are spared. Each patient received 2 GMA sessions/week, up to 10 sessions. One session was 60–90 min at 30 mL/min.

### Assessing ulcerative colitis severity

Clinical activity was assessed using the Lichtiger index.^24^ Lichtiger index cutoff values of 11 or higher indicated severe symptoms, 4–10 indicated moderate symptoms, and values of 3 or lower represented remission. Clinical improvement was defined as a score reduction of 50% or more from the baseline.

Prednisolone (PSL) resistance was defined as no clear improvement within 1–2 weeks, despite appropriate treatment with steroids. PSL dependence refers to a situation in which the PSL dose is reduced, leading to exacerbation or relapse and a difficult withdrawal.^3^, ^23^

### Assessment of disease activity and efficacy

For this study, the following information was analyzed based on medical records: gender, age, disease extent, clinical course classification, disease duration, remission‐inducing drugs, concomitant local therapy, PSL dependence, PSL resistance, and immunomodulator (IM) intolerance. The Lichtiger index at the beginning of remission induction and the Lichtiger index at the time of GMA induction were measured and recorded. The success rate of remission induction and the time necessary for remission induction were investigated.

### Statistical analysis

Statistical analyses were conducted using software (IBM SPSS Statistics 28; SPSS Inc., Chicago, IL, USA). Pearson's *χ* ^2^ test or Fisher's exact test was used to compare gender, disease extent, clinical course classification, concomitant local therapy, PSL dependence, PSL resistance, IM intolerance, Lichtiger index at the beginning of remission induction, Lichtiger index at the time of GMA induction, and successful remission induction. Student's *t*‐test or Mann–Whitney *U* test was used to compare mean age, mean disease duration, number of GMA procedures, and time from the beginning of remission induction to the end point. Multiple logistic regression and analysis of covariance were applied to analyze differences of the remission induction rate and mean time to remission induction in the early and late GMA combination groups. Potential confounders were items with a *P* value of 0.2 or less in univariate analysis. In addition, a propensity score was calculated using sex, age, type of remission induction therapy, severity at starting GMA, and severity at starting remission induction therapy, and early GMA combination group matched 1:1 to the late GMA combination group with caliber of 0.25 times the standard deviation of the propensity score. Finally, 13 pairs of the early and late GMA combination group were analyzed as sensitivity analysis. Results for which *P* < 0.05 were inferred as significant. A statistician evaluated the statistics calculated for this study.

## Results

### Success rate of remission induction achieved with GMA combination

**Figure 2:**
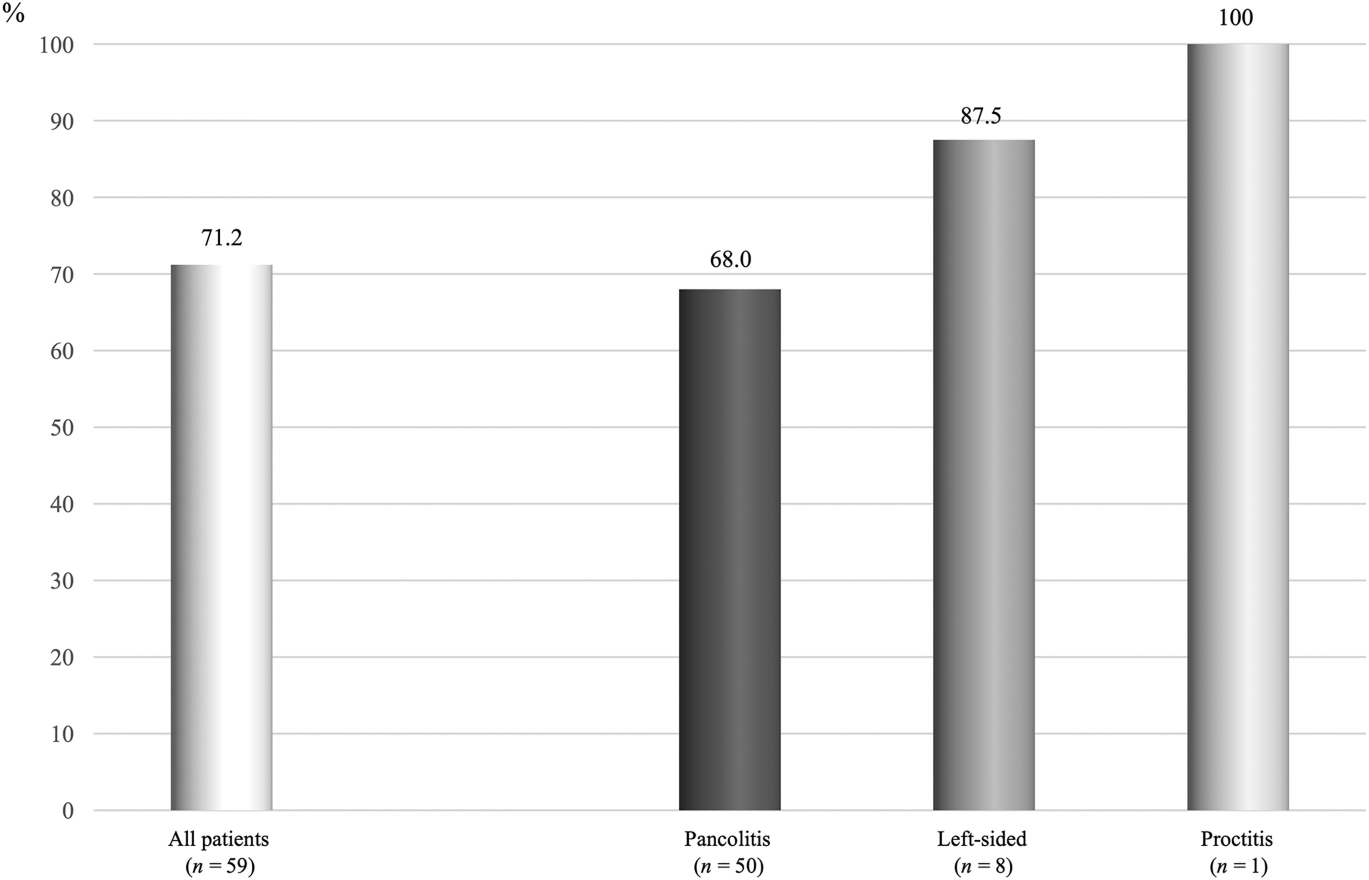
Remission induction rate by disease extent.

Table 1 presents background characteristics of the 59 study patients. According to the extent of the disease, proctitis type accounted for 1.7% of the patients (*n* = 1), left‐sided type for 13.6% (*n* = 8), and pancolitis type for 84.7% (*n* = 50). The median disease duration was 34 (1–462) months. The median Lichtiger index at the beginning of remission induction therapy was 13 (5–19). In addition, the median Lichtiger index at the beginning of GMA was 8 (4–15). Overall, remission induction was achieved in 71.2% (42/59) of patients. The remission induction rate by the disease extent was 68.0% (34/50) for pancolitis type, 87.5% (7/8) for left‐sided type, and 100% (1/1) for proctitis type (Fig. 2).

### Treatment success rates and time to induction of remission of the early GMA combination group and late GMA combination group

**Figure 3:**
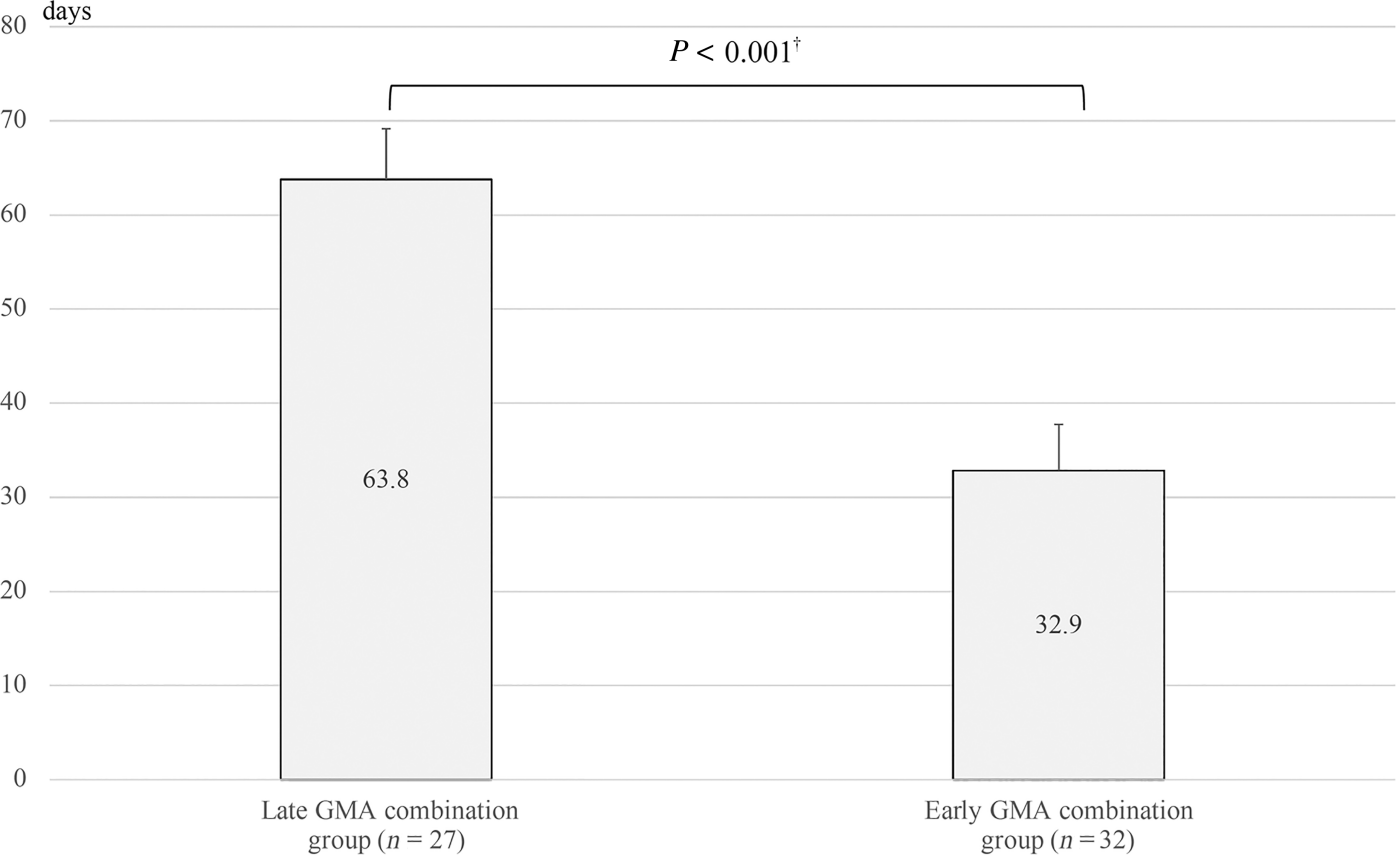
Mean time to remission induction of early and late granulocyte and monocyte adsorption (GMA) combination groups. Time to remission was compared using median with range. ^†^Using Mann–Whitney *U* test.

Table 2 presents the respective baseline characteristics of the early and late GMA combination group. No significant difference was found between the early and late GMA combination groups for gender, disease extent, clinical course classification, disease duration, concomitant local therapy, PSL dependence, PSL resistance, 5‐aminosalicylic acid intolerance, IM intolerance, Lichtiger index at the beginning of remission induction, or Lichtiger index at the beginning of GMA induction. Median age and remission induction therapy using PSL were significantly higher in the late GMA combination group. Of the 32 patients in the early GMA combination group, 24 achieved successful induction of remission. Furthermore, of the 27 patients in the late GMA combination group, 18 achieved successful induction of remission, with no significant difference in the induction rate (*P* = 0.481) (Table 2). The median time to induction of remission in the early GMA combination group was 30 (7–63) days, whereas the median time to induction of remission in the late GMA combination group was 63 (19–188) days, representing a significant difference between the two groups (*P* < 0.001) (Table 2). Figure 3 presents the results obtained using analysis of covariance, with mean times adjusted for age, sex, and PSL dependence for which the *P* value was less than 0.2.

**Table 2:** Patient characteristics of the early granulocyte and monocyte adsorption (GMA) combination group and the late GMA combination group.

Characteristics (*n* = 59)	Early GMA combination group (*n* = 32)	Late GMA combination group (*n* = 27)	*P* value^†^
Age (years)	31 (18–67)	43 (22–75)	0.005
Sex (male)	56.3% (*n* = 18)	51.9% (*n* = 14)	0.735
Extent of disease			0.405
Proctitis type	3.1% (*n* = 1)	0% (*n* = 0)	
Left‐sided type	9.4% (*n* = 3)	18.5% (*n* = 5)	
Pancolitis type	87.5% (*n* = 28)	81.5% (*n* = 22)	
Clinical course classification			0.687
First attack type	9.4% (*n* = 3)	3.7% (*n* = 1)	
Relapse‐remitting type	87.5% (*n* = 28)	92.6% (*n* = 25)	
Chronic continuous type	3.1% (*n* = 1)	3.7% (*n* = 1)	
Disease duration (months)	31 (1–282)	45 (2–462)	0.538^‡^
Remission induction therapy using PSL	40.6% (*n* = 13)	81.5% (*n* = 22)	0.001
Concomitant local therapy	25.0% (*n* = 8)	29.6% (*n* = 8)	0.690
PSL dependence	28.1% (*n* = 9)	44.4% (*n* = 12)	0.192
PSL resistance	18.8% (*n* = 6)	25.9% (*n* = 7)	0.508
5‐Aminosalicylic acid intolerance	0% (*n* = 0)	3.7% (*n* = 1)	0.458^‡^
Immunomodulator intolerance	18.8% (*n* = 6)	14.8% (*n* = 4)	0.741^‡^
Lichtiger index at the beginning of remission induction	13 (6–17)	12 (5–19)	0.261^‡^
Lichtiger index at the beginning of GMA induction	8 (5–14)	9 (4–15)	0.447^‡^
Remission induction rate	75% (*n* = 24)	66.7% (n = 18)	0.481
Median time to remission induction (days)	30 (7–63)	63 (19–188)	<0.001^‡^

^†^ Using the student's *t*‐test or the Chi‐squared test.

^‡^ Using the Mann–Whiney *U* test or the Fisher's exact test.

PSL, prednisolone.

Propensity score matching was applied to analyze the matched backgrounds of the early and late GMA combination groups. A sensitivity analysis was performed for the 13 groups in the propensity score matching (Table 3). Although the times to induction of remission were not significantly different because of the small number of cases, 32 days was found for the early group and 63 days for the late group, supporting the results of the overall analysis.

**Table 3:** Sensitivity analysis of the early granulocyte and monocyte adsorption (GMA) combination group and the late GMA combination group.

Characteristics (*n* = 26)	Early GMA combination group (*n* = 13)	Late GMA combination group (*n* = 13)	*P* value^†^
With a propensity score			
Age (years)	32 (18–67)	43 (28–75)	0.479
Sex (male)	61.5% (*n* = 8)	46.2% (*n* = 6)	0.431
Remission induction therapy using PSL	61.5% (*n* = 8)	84.6% (*n* = 11)	0.378
Lichtiger index at the beginning of GMA induction	8 (5–14)	9 (5–15)	0.650
Lichtiger index at the beginning of remission induction	14 (8–16)	12 (9–19)	0.650
Without a propensity score			
Remission induction rate	92.3 (*n* = 12)	61.5 (*n* = 8)	0.160
Median time to remission induction	32 (21–63)	63 (19–106)	0.091

^†^ Using the Mann–Whiney *U* test or chi‐squared test.

PSL, prednisolone.

### Safety profile

A few AEs were reported in the GMA: transient fever, dizziness, and light‐headedness developed toward the end of a GMA session. These events remitted within 3 h with no medication. No severe adverse event associated with GMA was observed during this study.

## Discussion

As a remission induction therapy for UC, GMA was introduced in the early 2000s. In recent years, reports of the relevant literature have described not only the efficacy of GMA monotherapy but also that additional therapy of GMA is effective in loss‐of‐response cases with infliximab and nonresponse or loss‐of‐response cases with vedolizumab.^25^, ^26^ Combination therapy with adalimumab and GMA was effective in resistance and dependence cases of corticosteroid or calcineurin inhibitor.^27^ Combination therapy is also being investigated as a treatment option, particularly considering the aspects of safety and efficacy.

Rapid remission induction is important for treating active UC. Clinical practice guidelines in various countries recommend that remission induction therapy with corticosteroids in moderate to severe UC be evaluated at 3–14 days.^3^, ^23^, ^28^, ^29^, ^30^ Actually, GMA is a treatment that adsorbs and removes activated granulocytes, monocytes, and macrophages.^31^, ^32^ Adsorbed leukocytes cause local inflammation‐like reactions such as reactive oxygen species release and degranulation in the column through an opsonin effect‐like mechanism. Inactivated leukocytes pass through the column without adsorption. Subsequently, leukocytes that pass through the column are exposed to reactive oxygen species, leading to decreased expression of adhesion molecules, decreased production of inflammatory cytokines, and increased production of anti‐inflammatory cytokines.^33^, ^34^ Early induction of GMA might prevent chronic inflammation. This study demonstrated a better treatment response by the group of patients who had been administered additional GMA after early determination of insufficient response to initial treatment. Moreover, the results demonstrated the importance of considering addition of GMA as soon as possible when the initial remission induction therapy is apparently inadequate, rather than easily changing the agent.

Regarding AEs, GMA safety has been reported not only as a monotherapy but also when used in combination therapy. Yokoyama et al. reported that no severe AE related to GMA was observed in 16 patients receiving GMA with infliximab.^25^ For this study, most patients were treated with GMA in combination with a corticosteroid or calcineurin inhibitor. The GMA safety was a major factor in the decision to use combination therapy. Moreover, AEs related to GMA combination therapy were not those judged to be attributable to the initial agent. Therefore, these results are evidence indicating safety for additional or combination therapy with GMA.

For remission induction therapy for UC, the major clinical question is how to proceed with subsequent treatment if the initial agent administered for remission induction is regarded as only partially responsive. If corticosteroid induction therapy is only partially effective, then one strategy is to switch to calcineurin inhibitor or anti‐cytokine therapy such as anti‐TNFα antibody.^3^, ^23^ However, one must consider whether adding a new agent to the treatment of patients with residual corticosteroid efficacy is truly safe and consider the possibility that an agent used as a second‐ or third‐line treatment is ineffective. If remission induction therapy with calcineurin inhibitors or anti‐cytokine therapy is regarded as inadequate, then it is necessary to add or change immunosuppressant agents to achieve remission induction, leading to an increase of two, three, or more immunosuppressant agents. In these situations, it is also necessary to prevent the development of infectious diseases, including cytomegalovirus and tuberculosis.^35^, ^36^ Continued treatment with calcineurin inhibitors or anti‐cytokine therapy does not guarantee slow remission induction. In fact, it prolongs the active phase of the disease. The results of this study, which indicated a remission induction rate of 71.2% with the addition of GMA, demonstrate the value of considering GMA as a new treatment option for patients who show partial response to such remission induction therapy. By contrast, differences in the initial treatment agent might affect the efficacy of GMA combinations in patients with UC. Furthermore, although GMA is certainly a safe treatment, some patients cannot be treated because of difficulties with blood access.

For administration of various remission maintenance agents after successful remission induction, the overall steroid‐free remission maintenance rate after 52 weeks was found to be 73.2%. A more or less favorable mid‐term prognosis was achieved. Recent reports have described that GMA is expected to be effective as a remission maintenance therapy.^37^ For this study, GMA was used as remission induction therapy in accordance with the conventional treatment strategy. After the prescribed number of GMA cycles, 5‐ASA, IM, and biological agents, which were used earlier for remission induction, were used for the maintenance of remission. For future studies, it might be valuable to consider new treatment strategies such as maintenance of remission with GMA.

As for limitations of the current study, this study was conducted as a single‐center, retrospective study. Therefore, the treatment and severity of disease in the target patients might vary, rendering accurate assessment of additional GMA potentially difficult. The differences in initial treatment might affect the efficacy of GMA combinations in patients with ulcerative colitis. Nevertheless, this study found no difference in baseline characteristics between the early and late GMA combination groups except for median age and remission induction therapy using PSL. Second, the number of cases was small. In particular, in propensity score matching, 13 cases in each group were analyzed. However, no earlier report of the relevant literature has described a study similar to this one. Although the number of cases was small, these results might contribute to the formulation of a treatment plan that is able to incorporate GMA into remission induction therapy. Multiple‐center prospective studies with a greater number of cases must be conducted to accumulate definitive evidence. Third, the possibility exists that some patients achieved remission with drug therapy alone. However, the Lichtiger index was high in both groups at the time of GMA introduction. The subsequent rapid and high remission induction rate suggests that GMA addition was beneficial and suggests that it was a reasonable treatment. Fourth, clinical remission was used as an outcome because neither endoscopic nor histopathological evaluation was possible after remission induction. For additional objective evaluation, future studies must evaluate the degree of improvement based on endoscopic findings and imaging findings, including histopathology.

In conclusion, the results presented herein suggest that early addition of GMA might lead to earlier remission in patients who have had an inadequate response to remission induction therapy with corticosteroids, calcineurin inhibitors, and anti‐cytokine therapy.

## Data Availability

All data generated or analyzed during this study are included in this article. Further enquiries can be directed to the corresponding author.
